# Research on image recognition of three *Fritillaria cirrhosa* species based on deep learning

**DOI:** 10.1038/s41598-023-46191-z

**Published:** 2023-11-09

**Authors:** Yuxiu Chen, Yuyan Li, Sheng Zhang

**Affiliations:** 1 Hunan Food and Drug Vocational College, Changsha, 410208 China; 2 College of Material Science and Engineering, Central South University of Forestry and Technology, Changsha, 410004 China

**Keywords:** Image processing, Machine learning, Network topology

## Abstract

Based on the deep learning method, a network model that can quickly and accurately identify the species of *Fritillaria cirrhosa* species was constructed. The learning method based on deep residual convolutional neural network was used to input the unprocessed original image directly as input, and the features of the image were extracted through convolution and pooling operations. On this basis, the ResNet34 model was improved, and the additional fully connected layer was added in front of the Softmax classifier to improve the learning ability of the network model. Total of 3915 images of three kinds of *Fritillaria cirrhosa* were used as data sources for the experiments, among which 160 images of each type were randomly selected to form the validation set. The final training set recognition accuracy rate was 95.8%, the validation set accuracy rate reached 92.3%, and the test set accuracy rate was 88.7%. The image recognition method of *Fritillaria cirrhosa* based on deep learning proposed in this paper is effective and feasible, which can quickly and accurately identify the species of *Fritillaria cirrhosa* species, and provides a new idea for the intelligent recognition of Chinese medicinal materials.

## Introduction

As a traditional Chinese medicine with a long medicinal history, *Fritillaria cirrhosa* has the functions of clearing heat and moistening lung, relieving cough and reducing sputum, and resolving carbuncle and expulsing boil. It is listed as a medium-grade herb in the “Shennong’s Classic of Material Medical” and has the reputation of being a “holy medicine for relieving cough”^1^. It is derived from the dried bulbs of the genus *Fritillaria* (Liliaceae), such as *Fritillaria unibracteata* Hsiao et K. C. Hsia, *Fritillaria cirrhosa* D. Don, *Fritillaria przewalskii* Maxim., *Fritillaria delavayi* Franch., *Fritillaria taipaiensis* P. Y. Li, and *Fritillaria unibracteata* Hsiao et K. C. Hsia var. *Wabuensis* (S. Y. Tanget S. C. Yue) Z. D. Liu, S. Wang et S. C. Chen^2^. According to the different properties, the quality of *Fritillaria cirrhosa* can be divided into Songbei, Qingbei, Lubei and cultivated products from high to low. There are more than 200 kinds of Chinese patent drug produced with *Fritillaria cirrhosa* as the main raw material, and the demand in the market is high. Moreover, the growth conditions of *Fritillaria cirrhosa* are harsh, and excessive harvesting has sharply reduced its resources, leading to a continuous rise in price^3^. Among them, the price of Songbei is the highest, followed by Qingbei, and Lubei is the lowest, resulting in the phenomenon of inferior products being sold as better ones in the market. Besides the price, there are also some differences in medicinal properties of different *Fritillaria cirrhosa*. Therefore, the correct identification of *Fritillaria cirrhosa* is not only related to the economic interests of consumers, but also to the quality and clinical efficacy of Chinese cut crude drugs^4^. How to quickly and accurately identify the species of *Fritillaria cirrhosa* is a problem and challenge faced by primary medicine practitioners at present.

The empirical identification, thin-layer chromatography and DNA labeling methods were used to identify *Fritillaria cirrhosa* in the Chinese Pharmacopoeia, 2020 edition I. Currently, the identification methods of *Fritillaria cirrhosa* can be divided into the following four categories: (1) Empirical identification method, judging based on the appearance characteristics, such as the shape, color and size of *Fritillaria cirrhosa*^5^. This kind of method is easy to operate but requires high personnel, and the identifier needs special training to accumulate extensive experience in pharmaceutical identification. There are individual differences, and the recognition results are easily affected by sensory sensitivity, subjectivity, fatigue and other factors. (2) Physicochemical method, recognizing based on the characteristic chemical composition of *Fritillaria cirrhosa*, such as thin-layer chromatography^6^, spectrophotometry^7^, thermogravimetric analysis^8^, high performance liquid chromatography^9^, fluorescence spectrometry^10^, electrospray mass spectrometry^11^, electron tongue and electron nose^12^. The results of these methods are stable and reliable, but they are inefficient, time-consuming, and require expensive instruments and sophisticated operations, which are difficult to popularize in grassroots units. (3) Biological method, classifying based on characteristic protein or DNA of *Fritillaria cirrhosa*, such as protein mapping analysis, DNA labeling and molecular probe technology^13,14^. This type of method is highly sensitive and specific, but require high analytical equipment and operating environment. (4) Image processing and pattern recognition method, which uses computer technology and mathematical methods to process and analyze images of *Fritillaria cirrhosa*, and classify them according to features^15^. Although empirical identification is the most widely used and classic method based on appearance, the contradiction between “high requirements” and “labor cost” has become more and more obvious as the identification requirements of *Fritillaria cirrhosa* have increased, especially as the frequency of identification continues to increase. Therefore, the image processing and pattern recognition with automatic identification function is considered to be one of the hot research directions of *Fritillaria cirrhosa* species recognition in the future.

Automated identification through image processing is an important approach to achieving automation in the identification of *Fritillaria cirrhosa*. Wang et al. extracted eight features such as multi-scale wavelets and fractal dimension, and used discriminant analysis for automatic identification of *Fritillaria cirrhosa* powder micrographs^16^. Liu et al. proposed an edge detection algorithm based on multi-scale wavelet transform to accurately and effectively extract the edges of the target information in *Fritillaria cirrhosa* micrographs, and then realize the automatic classification of *Fritillaria cirrhosa*^17^. Liu et al. used transfer learning to train a model, combined with EfficientDet-B0 target detection network and *k*-means clustering algorithm to the achieve automatic identification of three species of *Fritillaria cirrhosa*^18^. These methods not only require the use of image segmentation algorithms to assist in localization of *Fritillaria cirrhosa*, but also need to manually design the relevant extraction features, which is difficult to meet the requirements of rapid automatic recognition. Thanks to its reasonable network structure, the deep convolutional neural networks are able to automatically learn image features and achieve end-to-end classification results output while balancing efficiency and accuracy.

At present, there are few researches using deep learning method for the identification of *Fritillaria cirrhosa*. Hu et al. classified the *Fritillaria cirrhosa* dataset based on the deep learning framework SE-DPU, but did not provide the recognition accuracy and related misjudgment results, nor did they describe the structure and relevant parameters of the neural network used^15^. Therefore, this paper proposes a deep learning-based image recognition method to classify images of three types of *Fritillaria cirrhosa*, and establishes a ResNet34 model suitable, which is of great significance for quickly and accurately identifying *Fritillaria cirrhosa* species.

## Materials and methods

### Material preparation

**Figure 1:**
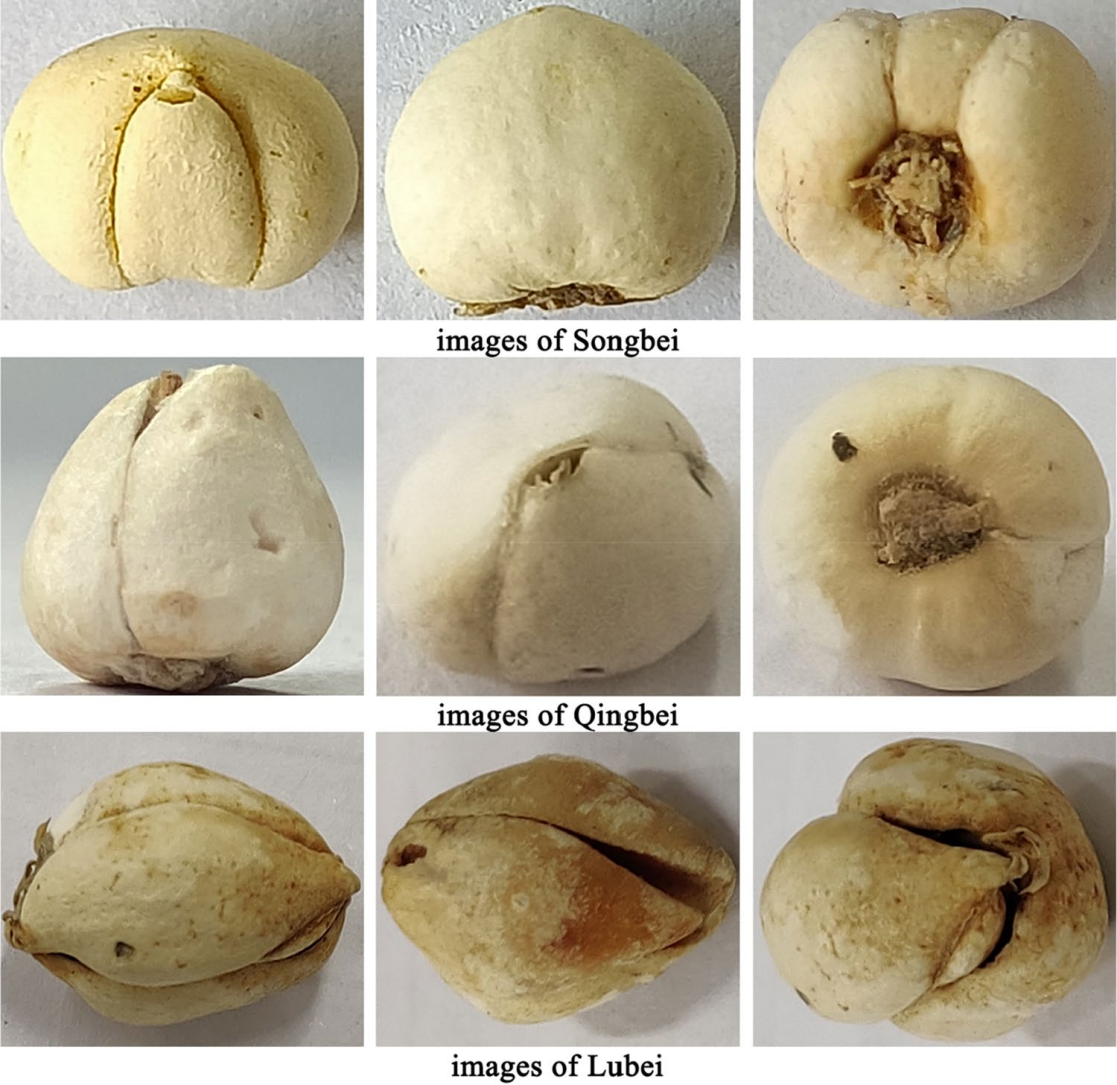
Examples of images of Songbei, Qingbei and Lubei.

In this study, three commercial specifications of the traditional Chinese medicine *Fritillaria cirrhosa* (Songbei, Qingbei and Lubei) were selected as the research objects. It comes from Sichuan Province Origin Medicinal Plant Planting Co., Ltd., with batch numbers of 20170228, 20170306 and 20170309, respectively. The samples were identified as Songbei, Qingbei and Lubei by Professor Ribao Zhou from Hunan University of Chinese Medicine. To make the experimental images more representative of the natural circulation state of the market and reflect the randomness of the sample selection process, different photographers, light intensities, shooting equipment (ordinary smartphones) and shooting angles were selected to collect images of *Fritillaria cirrhosa*. A simple preprocessing of the image was performed using python language without destroying the original information of the image, and the area of 224 × 224 pixels was intercepted in its middle part. After eliminating the relevant substandard images, a total of 3915 images of *Fritillaria cirrhosa* were obtained, including 1274 images of Songbei, 1316 images of Qingbei and 1325 images of Lubei. 1054 images of Songbei, 1102 images of Qingbei and 1107 images of Lubei were randomly selected to form the training set, and 160 images of each specification of *Fritillaria cirrhosa* were randomly selected to form the validation set. Finally, the remaining 60 images of Songbei, 54 images of Qingbei and 58 images of Lubei were used as the test set. The images of three specifications of *Fritillaria cirrhosa* were shown in Fig. 1.

### Experimental method

**Figure 2:**
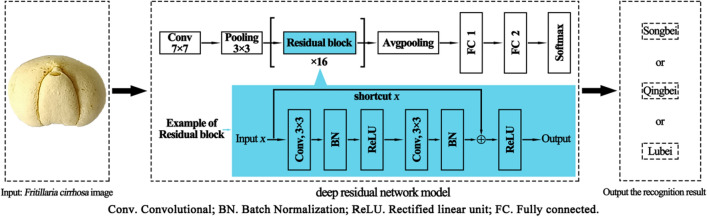
Flow chart of automatic identification of *Fritillaria cirrhosa* based on deep residual network model.

In this study, an automatic classification of *Fritillaria cirrhosa* images was proposed by constructing a deep residual convolutional neural network, and the recognition process was shown in Fig. 2. Inputting an image of *Fritillaria cirrhosa*, processing it with the improved ResNet34 deep residual network, and then outputting the *Fritillaria cirrhosa* species. The deep residual network model contains four residual units with 3, 4, 6 and 3 corresponding residual blocks, for a total of 32 layers, plus a Conv 7 × 7 convolution layer and two fully connected layers, for total of 35 layers (excluding the pooling layer). Each residual block includes convolution, batch normalization, activation function, and directly connected shortcut across layers. After being processed by the residual unit, the size of the feature map was adjusted by the downsampling effect of the avgpooling layer and finally connected to the fully connected layer. The Softmax classifier was used with three output categories. Compared with the conventional ResNet34 model, an additional fully connected layer was added in front of Softmax to improve the learning ability of the network model.

### Convolutional neural network

Convolutional neural network (CNN), a concept introduced by Hubel et al.^19^ in the 1960s, was a major application of deep learning in the field of image recognition. In recent years, a quantity of scholars have improved and optimized the original CNN model, making it become one of the most widely used methods in the field of image processing. With AlexNet winning the ImageNet competition in 2012, CNN officially stepped into a period of rapid development, and many deeper CNNs have been proposed one after another. With the emergence of VGG network, a deeper network structure and smaller convolutional kernels were adopted to improve the model performance, achieving excellent results in both image classification and localization tasks. However, as the network layers deepen, it will gradually lose the detailed feature information of the shallow layers, making training more difficult and easily triggering the degradation problem of vanishing gradient^20^.

To address this issue, He et al. from Microsoft Labs put forward the ResNet residual network in 2015^21^. By introducing the idea of residual learning into CNN, the feature information of different layers can be transmitted to each other, which effectively alleviates the problem of vanishing gradient in deep network and guarantees its image recognition performance. According to the number of network layers, ResNet can be mainly divided into five types, namely ResNet18, ResNet34, ResNet50, ResNet101 and ResNet152, where the number indicates the number of network layers. With the increase of network layers, the relevant parameters and computational complexity also increase, and the training speed of the network will slow down, which may lead to a decrease in recognition accuracy. If the number of network layers is too small, the feature expression capability will be lacking, which will affect the accuracy of model recognition. Zhou et al. investigated the ResNet models with different network layers for the classification of traditional Tibetan medicine plant and found that ResNet34 residual network had the highest recognition accuracy and precision mean value^22^. On the basis of taking certain feature expression ability into account, to better improve the network training speed and control the error rate, the ResNet34 residual network model was selected as the classification framework for *Fritillaria cirrhosa* images in this paper.

The ResNet34 residual network primarily contains three different layers, namely convolutional layer, pooling layer and fully connected layer. The convolutional layer was often used to capture local information of images and consists of feature maps. Since there are many convolutional kernels in the convolutional layer, multiple feature maps will be generated after the convolution calculation. The convolution formula was as follows^23^:

1 $${X}_{j}^{l}=f\left({\sum }_{i\in {M}_{j}}{X}_{i}^{l-1}\times {k}_{ij}^{l}+{b}_{j}^{l}\right),$$

where ${X}_{j}^{l}$ is the *j*th feature map of the *l*th convolutional layer, *f*( ) refers to the activation function of the neuron, ${M}_{j}$ represents the combination of input feature maps in the *l* − 1 layer, ${k}_{ij}^{l}$ denotes the convolutional kernel matrix between the *i*th input and the *j*th output feature maps, ${b}_{j}^{l}$ means the bias matrix corresponding to the *j*th feature map, *l* suggests the *l*th convolutional layer, *j* represents the *j*th feature, and *i* denotes the *i*th network layer.

The pooling layer, also known as the sampling layer, can obtain features with spatial invariance by reducing the resolution of the output feature map of the previous convolutional layer^24^. It reduces the number of parameters and improves the computational efficiency while retaining the useful information in the image. Common pooling methods can be classified into the following four categories: mean pooling, minimum pooling, maximum pooling, and random pooling^25^. The sampling formula was as follows:

2 $${X}_{j}^{l}=f\left({W}^{l}{X}^{l-1}+{b}^{l}\right),$$

where down( ) means the down-sampling function; *ꞵ* represents the output feature bias, and the value is determined according to the actual situation.

The fully connected layer is mainly used to classify the image features obtained through a series of processing such as convolution and pooling. It can integrate the local information of feature extraction while reduce the dimension of image representation, so as to obtain the global image recognition category. Each neuron of the fully connected layer was connected with the corresponding neuron of the next layer, generally represented as follows:

3 $${X}_{j}^{l}=f\left({W}^{l}{X}^{l-1}+{b}^{l}\right),$$

where ${W}^{l}$ refers to the weight matrix, ${b}^{l}$ denotes the bias vector.

### Parameter optimization algorithm

The Randomized Search Method (RSM) is a hyperparametric search algorithm proposed by Yoshua Bengio and James Bergstra in 2012^26^. It selects a random value of each hyperparameter for random combination by random sampling. It does not go through all the parameter combinations, thus greatly reducing the computational effort of parameter search, reducing the optimization time, and then improving the model performance. As a commonly used hyperparameter optimization method, random search has an absolute advantage over grid search and manual search in reducing the search time while taking the accuracy of the model into account.

### Activation function

To endow the neural network with hierarchical nonlinear learning capability, an activation function was introduced in its structure. Compared with the common saturated activation functions such as sigmod and tanh, ReLU solves the problem of vanishing gradient well and speeds up the network training because of its sparsity and non-saturated form^27^. In this study, ReLU was used as the activation function with the following equation:

4 $$f\left(x\right)=\left\{ \begin{array}{c}x, x\ge 0\\ 0, x<0\end{array}\right.,$$

### Batch normalization

Batch normalization (BN) is another important technique in ResNet34 network to solve the problem of vanishing gradient in addition to the residual structure. In the process of model training, the data output from the convolutional layer is batch normalized to make it conforms to the normal distribution law with mean value of 0 and variance of 1, so that the input values of the activation function fall in the linear region where the nonlinear function is more sensitive to the input. In this way, a slight change in the input will also lead to a significant change in the loss function, which increases the gradient and effectively alleviates the problem of vanishing gradient^20^. Moreover, the increased gradient also means that the model learning converges faster, which can improve the training speed and the network generalization ability. While reducing the risk of network overfitting, it also improves the recognition accuracy. The algorithm is as follows^20^:

*Input* samples {*x*_1_*, x*_2_*, *…,* x*_*m*_} in a data block, with a total of *m* samples, available for learning parameters *γ* and *β*.

*Output* batch normalized data {${y}_{i}={BN}_{\gamma ,\beta }\left({x}_{i}\right)$}.

1. Calculate the mean value of input sample ${\mu }_{B}$: ${\mu }_{B}=\frac{1}{m}\sum_{i=1}^{m}{x}_{i}$.
2. Calculate the variance of the input sample ${\sigma }_{B}^{2}$: ${\sigma }_{B}^{2}=\frac{1}{m}\sum_{i=1}^{m}{\left({x}_{i}-{\mu }_{B}\right)}^{2}$.
3. Batch normalization of the current data: $\widehat{{x}_{i}}=\frac{{x}_{i}-{\mu }_{B}}{\sqrt{ {\sigma }_{B}^{2}+\varepsilon }}$.
4. Transformation of the batch normalized data by the available learning parameters *γ* and *β* (where *γ* has an initial value of 1 for adjusting the variance and *β* has an initial value of 0 for regulating the mean value; *γ* and *β* were continually updated and adjusted during the backpropagation learning process): ${y}_{i}\equiv {BN}_{\gamma ,\beta }\left({x}_{i}\right)=\gamma \widehat{{x}_{i}}+\beta$.

### Loss function

To measure the difference between the distribution learned by the network model and the real distribution, ResNet34 network adopts the cross entropy loss function. The resulting cross entropy loss function was convex and monotonic by weighted averaging of the cross entropy of the samples. The larger the loss, the larger the gradient, which improves the speed of optimization during backpropagation^28^. The formula is as follows:

5 $$\mathrm{Loss}=-{\sum }_{j}{p}_{ij}\mathrm{log}\left({q}_{ij}\right),$$

where ${p}_{ij}$ represents the true probability that the *i*th sample belongs to class *j*, ${q}_{ij}$ refers to the predicted probability that the *i*th sample belongs to class *j*, Loss indicates the output loss function value.

### Learning rate decay

When training a convolutional neural network, the performance of the gradient descent method needs to be guaranteed by continuously adjusting the learning rate to give the model a better learning ability. Learning rate decay refers to setting a larger learning rate at the early stage of training to make the network converge quickly, and setting a smaller learning rate at the later stage of training so that the network can converge better to achieve the optimal solution^29^. This article adopts a fixed step decay, setting the initial learning rate to 0.005, and multiplying the learning rate by the decay coefficient 0.9 every 10 epochs.

### Plant research statement

*Fritillaria cirrhosa* is not in the range of endangered plant and animal species and it also is not listed by IUCN as Vulnerable, Rare, Endangered, or Indeterminate. Our all methods were carried out in accordance with relevant guidelines, including the IUCN Policy Statement on Research Involving Species at Risk of Extinction and the Convention on the Trade in Endangered Species of Wild Fauna and Flora. *Fritillaria cirrhosa* was collected from legitimate Chinese herbal medicine markets in Changsha city under permit No. 20-3-39, in compliance with China’s Wild Plant Protection Law. It was stored at Hunan Food and Drug Vocational College, and identified by Yun Lin, an Associate Professor in Chinese medicinal plant identification.

## Results and analysis

The experimental environment of this study: a computer with hardware configuration of Intel(R) Core(TM) i5-9300H CPU @ 2.40 GHz and 16 GB RAM. The program was run on Windows 10 system and Pycharm code platform. The code involved in this experiment was written in python 3.8 programming language based on the Pytorch 3.8 framework.

### Training process

**Figure 3:**
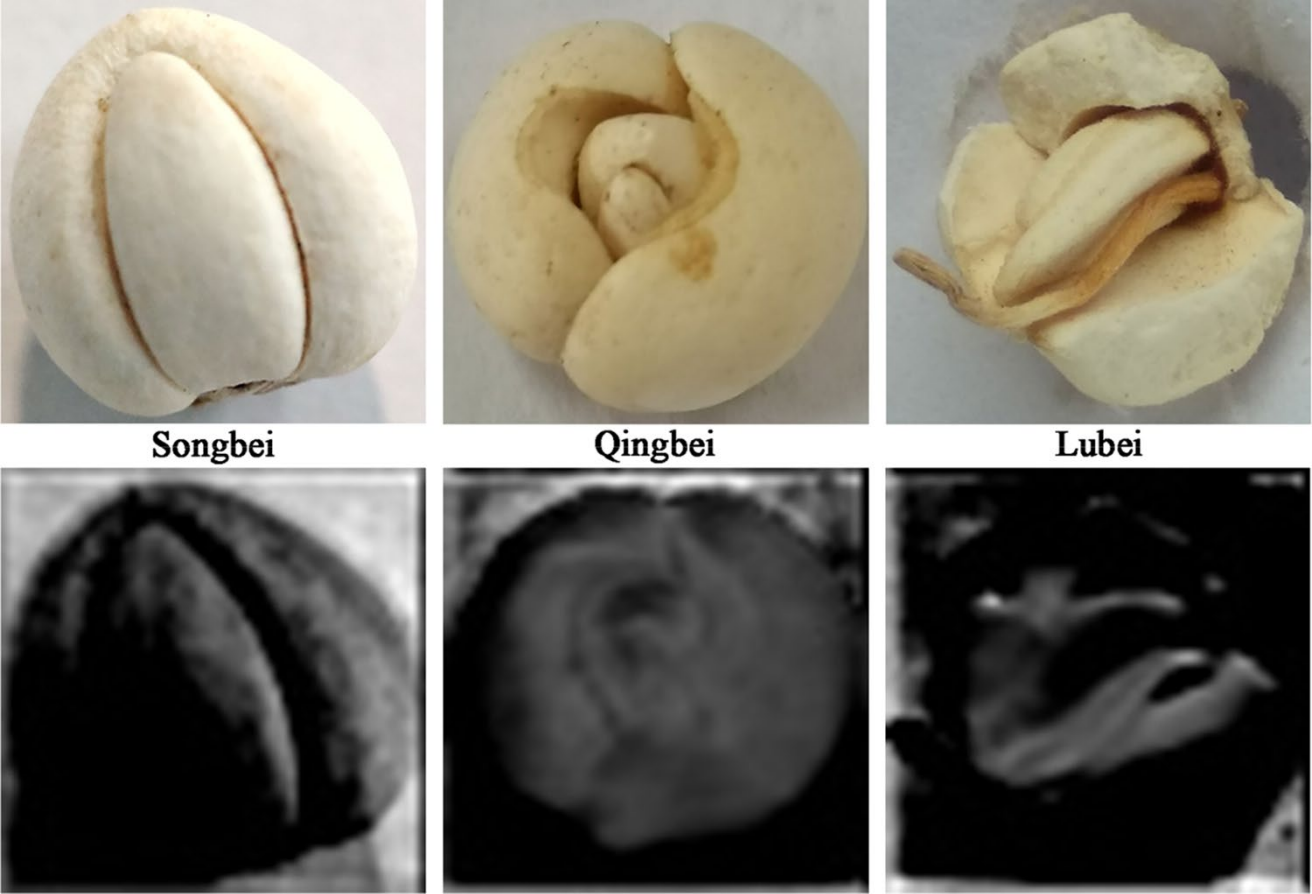
Feature maps visualization.

To better reveal the training process of the ResNet34 convolutional neural network model in this study, the feature maps extracted from the first convolutional layer corresponding to the input *Fritillaria cirrhosa* images are shown in Fig. 3. It is not difficult to find that the convolutional layers have well extracted the various features of the three kinds of *Fritillaria cirrhosa* images, and basically retained all the information of the original images. With the increase of network layers, the image content that can be extracted by the convolutional layer will become more abstract, and the information that can be retained will be less. For the ResNet34 model, it can extract as many original image features as possible through layer-by-layer extraction, which is conducive to the subsequent training of the model and lays a good foundation for its high recognition accuracy.

**Figure 4:**
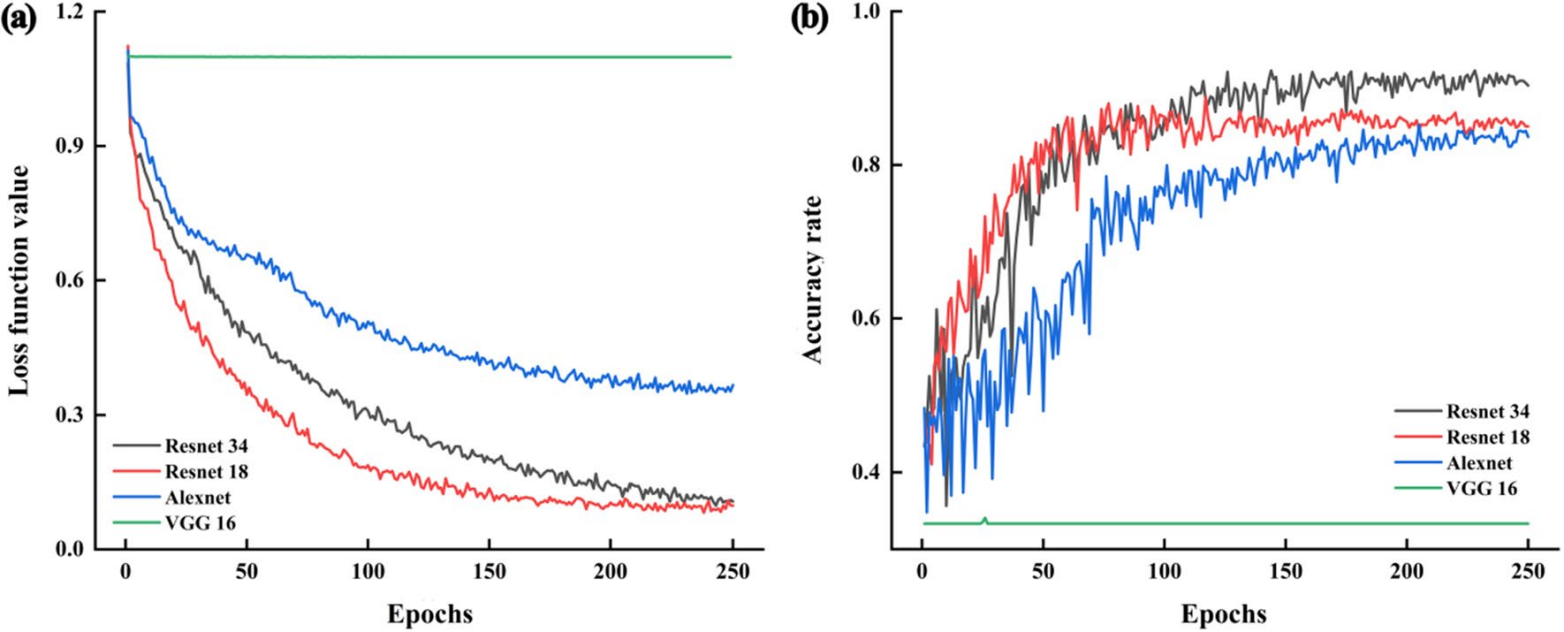
(**a**) Variation curve of loss function value; (**b**) variation curve of accuracy rate.

This study carried out a visualization analysis of the training process for four deep learning models: ResNet34, ResNet18, AlexNet and VGG16. All models were trained for 250 epochs, and the changes of loss function values of the training set and accuracy of the validation set with the number of iterations were recorded by visualization tools to ensure the normal operation of deep learning algorithm during the training process. The visualization results of the training process were shown in Fig. 4. The loss function value of the training set tends to be stable between 200 and 250 iterations while the accuracy of the verification set tends to be stable between 150 and 200 iterations. Based on the comprehensive changes of loss function values and accuracy, it was reasonable to set 250 epochs of model training. In Fig. 4a, the red curve was at the bottom, followed by the black curve. In Fig. 4b, the black curve was basically at the top, followed by the red curve. This indicated that the loss function value of ResNet34 decreased second only to ResNet18 and the accuracy rate increased the fastest, which means that the ResNet34 algorithm has better convergence. This may be because ResNet34 has more network layers, so its loss function value dropped slightly slower than ResNet18, but its recognition accuracy was higher. Compared with AlexNet and VGG16, the ResNet residual network exhibited faster loss function decline and accuracy increase during the training process.

### Model recognition results

The trained ResNet34 deep residual network model was used to test the recognition of a total of 480 images of three kinds of *Fritillaria cirrhosa* in the validation set, and the results were shown in Table 1. It can be found that the recognition accuracy of Lubei was the highest (98.7%), followed by that of Songbei (90.0%), and that of Qingbei was the lowest (88.3%), with an average recognition accuracy of 92.3%. The relatively low recognition accuracy of Songbei and Qingbei was due to the higher similarity of their captured images, which makes them more susceptible to misjudgment.

**Table 1:** Recognition results of three kinds of *Fritillaria cirrhosa* images.

*Fritillaria cirrhosa* species	Accuracy rate/%	Recall rate/%
Songbei	90.0	90.0
Qingbei	88.3	89.4
Lubei	98.7	97.5
Average value	92.3	92.3

The confusion matrix of the ResNet34 model’s recognition results of the validation set was shown in Table 2. It is not difficult to find that 15 images of Songbei were misjudged as Qingbei, and 16 images of Qingbei were misjudged as Songbei, with a high similarity between the two bottoms. One image of Qingbei was misjudged as Lubei, and four images of Lubei were misjudged as Qingbei, because both of which have similar scales. The scales of Songbei were one large and one small in the shape of “holding the moon in the arms”, with a closed top and flat bottom. The scales of Qingbei were of equal size in the shape of “Avalokitesvara sitting lotus”, with an opened top and flat bottom. The scales of Lubei were equal in size, with an opened top and sharp bottom. The Songbei was more recognizable from the scales and top angle, while the Lubei was more recognizable from the bottom. The images of Songbei and Qingbei taken from the bottom were highly similar and easy to misjudge, while the Qingbei and Lubei were highly similar and easy to misjudge from the perspective of scales. It can be seen that the shooting angle of *Fritillaria cirrhosa* images has a certain influence on the recognition results.

**Table 2:** Confusion matrix of discrimination results of three kinds of *Fritillaria cirrhosa* images.

*Fritillaria cirrhosa* species		Predicted value		
		Songbei	Qingbei	Lubei
True value	Songbei	144	15	1
	Qingbei	16	143	1
	Lubei	0	4	156

**Figure 5:**
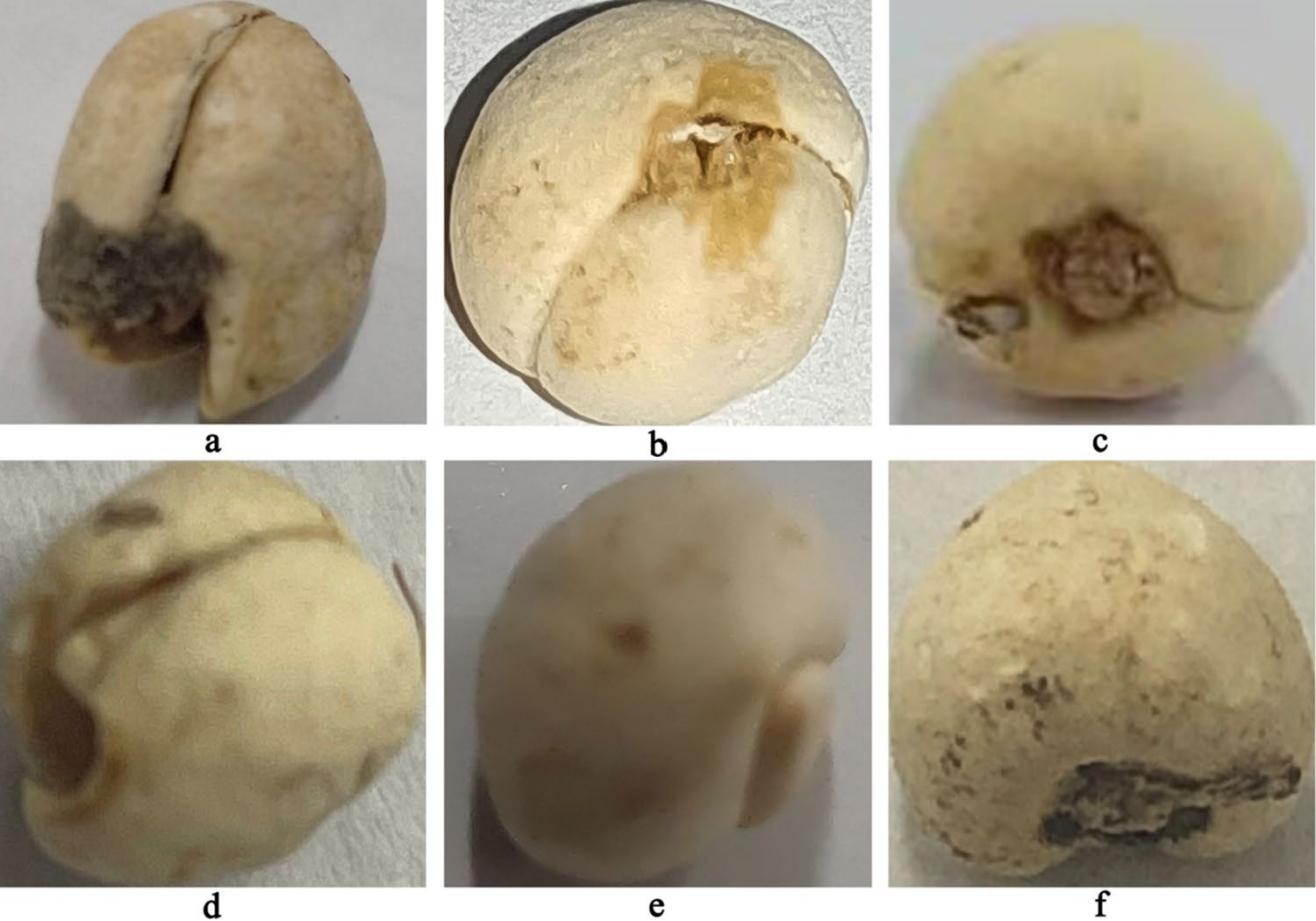
Partially misjudged fritillary images.

Some misclassified images were shown in Fig. 5. The Lubei was misjudged as Qingbei (Fig. 5a), which have similarity in appearance and morphology with an opened top and equal sized scales. The Qingbei was misjudged as Lubei (Fig. 5b) because the yellow–brown spot on the top of this image was similar to that of Lubei. Figure 5c,d were images of Qingbei, which were misjudged as Songbei due to their special shooting angles that caused the scales to be one large and one small. Figure 5e,f were images of Songbei, which were misjudged as Qingbei because they were taken from above the large scales and did not show the small scales, resulting in the possibility of equal scale sizes.

Furthermore, to evaluate the generalization performance of the model, the images in the test set were recognized, and the recognition results were presented in Table 3. In the test set, the recognition accuracy of Songbei was the highest (92.9%), followed by that of Lubei (92.6%), and that of Qingbei was the lowest (80.6%). This was consistent with the lowest recognition accuracy of Qingbei in the validation set, while the recognition accuracy of Songbei and Lubei was higher. The possible reason was that the identification degree of Songbei and Lubei was higher, and the number of samples in the test set was relatively small, which has some influence on the recognition results. The average recognition accuracy of test set was 88.7%, which was slightly lower than that of the validation set, but still maintained at a high level and has some practical application value. The confusion matrix of its recognition results was revealed in Table 4. Among them, 6 images of Songbei were misjudged as Qingbei and 2 images of Qingbei were misjudged as Songbei, 2 images of Songbei were misjudged as Lubei and 2 images of Libei were misjudged as Songbei, and 2 images of Qingbei were misjudged as Lubei and 6 images of Libei were misjudged as Qingbei. Both Songbei and Lubei were easily misjudged as Qingbei, which was consistent with the confusion matrix results of the validation set. In addition, the image recognition comparison of the improved ResNet34 from this work with other similar models was shown in Table 5. Obviously, the improved ResNet34 model demonstrates higher recognition accuracy and recall rate, making it highly valuable for practical applications.

**Table 3:** Test set recognition results.

*Fritillaria cirrhosa* species	Accuracy rate%	Recall rate/%	specificity/%
Songbei	92.9	86.7	96.4
Qingbei	80.6	92.6	89.8
Lubei	92.6	86.2	96.5
Average value	88.7	88.5	94.2

**Table 4:** Confusion matrix of discriminant results of test set.

*Fritillaria cirrhosa* species		Predicted value		
		Songbei	Qingbei	Lubei
True value	Songbei	52	6	2
	Qingbei	2	50	2
	Lubei	2	6	50

**Table 5:** Comparison of image recognition of *fritillaria cirrhosa* of similar models.

Model	Accuracy rate%	Recall rate/%	Ref.
Improved ResNet34	88.7	88.5	This work
EfficientDet-D0	83.0	71.5	^18^
EfficientDet-D0-K	85.3	79.8	^18^

### Comparison of deep learning models

**Figure 6:**
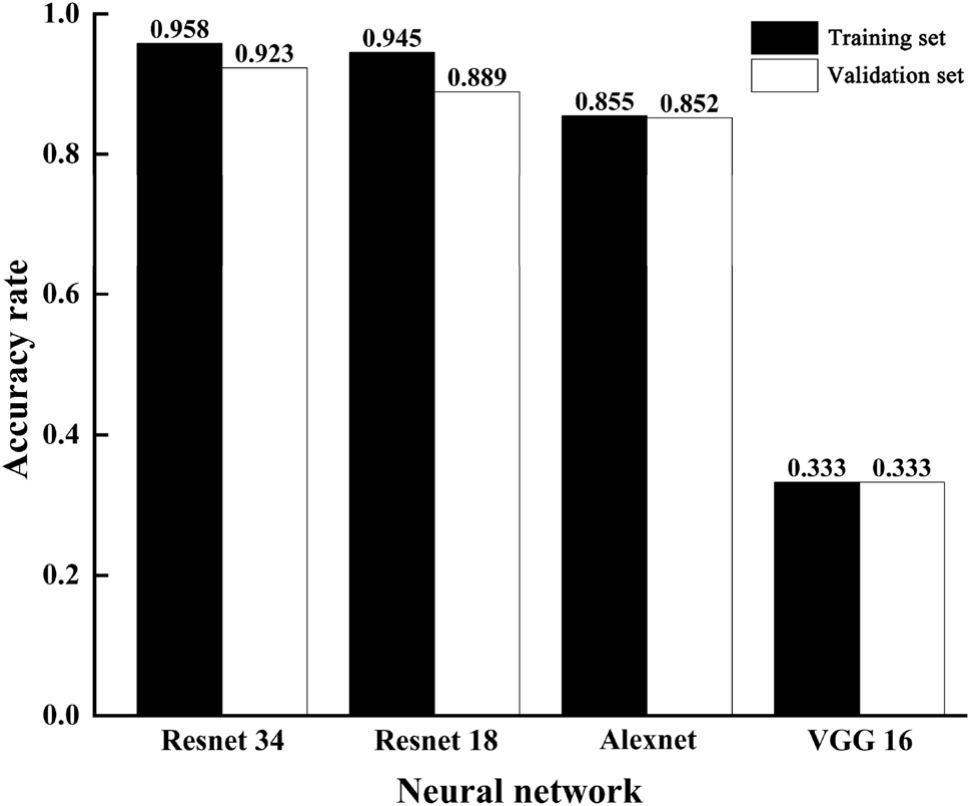
Experimental results with different convolutional neural networks.

To verify the feasibility of the improved ResNet34 network model proposed in this study, a comparative study was conducted with the classical ResNet18, Alexnet and VGG16 models in deep learning under the same computing environment, and the results were shown in Fig. 6. It can be seen that the improved ResNet34 had the highest recognition accuracy on both the training and validation sets, which were above 90%. ResNet18 had the second-highest recognition accuracy, while VGG16 had the lowest recognition accuracy of 33.3%. The residual blocks in the ResNet34 residual network adopt skip connections to bypass the input information to the output, so that the later layer can directly learn the residuals, which greatly reduces the learning difficulty while ensuring the information integrity. It increases the depth of the network to improve the accuracy while well alleviates the problem of vanishing gradient that exists in deep networks and avoiding model degradation. The VGG16 network model failed to learn the features of Qingbei and Songbei, so its training and validation sets were all recognized as Lubei. The possible reason for this is that its parameters are too large to be applicable for the recognition of *Fritillaria cirrhosa*. In this study, the recognition accuracy and generalization ability of the improved ResNet34 model were superior to the above three classical deep learning models. It is not difficult to find that the improved ResNet34 network can better extracted the features of *Fritillaria cirrhosa*, achieving the highest recognition accuracy. The proposed model in this research was effective and feasible.

## Conclusion and discussion

Based on deep learning methods, this study conducted classification and recognition research on the images of three kinds of *Fritillaria cirrhosa* on the market, including Songbei, Qingbei and Lubei, by establishing a residual convolutional neural network model. The main conclusions were as follows:

1. This study utilized the advantages of deep residual convolutional neural networks, which can automatically extract image features, avoiding the influence of human subjectivity caused by manual extraction of image features and excluding individual differences. Besides, its network structure was also suitable for processing a large quantities of sample data, with high recognition accuracy.
2. To improve the learning ability of the network model, this study added an additional fully connected layer before the Softmax classifier. To find the optimal solution, the fixed step decay method was used to regulate the learning rate. The visual analysis of the training process was carried out to determine the best iteration times of the model training and guarantee the recognition accuracy.
3. To verify the correctness of this research method, a comparative study was conducted with the classical ResNet18, Alexnet and VGG16 models in deep learning under the same computing environment. The recognition accuracy of this method was 92.3%, significantly higher than 88.9% of ResNet18, 85.2% of Alexnet and 33.3% of VGG16. It can be seen that this research method was feasible and effective.
4. In this study, a deep learning-based image recognition method was used to achieve rapid and accurate identification of *Fritillaria cirrhosa* species, which provides an application research foundation for developing mobile phone software or mini apps that can be used for online recognition of *Fritillaria cirrhosa*. It not only ensures the economic interests of consumers, but also guarantees the quality and clinical efficacy of Chinese cut crude drugs, which well alleviates the challenges faced by grassroots medical practitioners.

Although this research method had a high recognition accuracy and successfully classified the images of three kinds of *Fritillaria cirrhosa*, there were still areas for improvement. For example, the recognition accuracy of Songbei, which had the most obvious appearance features, was not as accurate as that of Lubei. Moreover, this study only conducted experiments on three kinds of *Fritillaria cirrhosa*, and whether it can be applied to the identification of other kinds of *Fritillaria cirrhosa* remains to be further investigated. Additionally, studying the microscopic image features of *Fritillaria cirrhosa* powder to broaden its practical applications will be a focus of future work.

## Data Availability

The datasets used and analysed during the current study available from the corresponding author on reasonable request.
